# A mother-child intervention program in adolescent mothers and their children to improve maternal sensitivity, child responsiveness and child development (the TeeMo study): study protocol for a randomized controlled trial

**DOI:** 10.1186/s13063-015-0747-5

**Published:** 2015-05-27

**Authors:** Christine Firk, Brigitte Dahmen, Christin Lehmann, Anke Niessen, Julia Koslowski, Geraldine Rauch, Reinhild Schwarte, Kerstin Stich, Kerstin Konrad, Beate Herpertz-Dahlmann

**Affiliations:** Department of Child and Adolescent Psychiatry, University Hospital RWTH Aachen, Neuenhofer Weg 21, 52074 Aachen, Germany; Institute of Neuroscience and Medicine (INM-3), Forschungszentrum Jülich, Leo-Brandt-Straße, 52425 Jülich, Germany; Institute of Medical Biometry and Informatics, University of Heidelberg, Im Neuenheimer Feld 305, 69120 Heidelberg, Germany

**Keywords:** Maternal sensitivity, Child development, Maltreatment, Adolescent mothers, STEEP, Video feedback

## Abstract

**Background:**

Children of adolescent mothers present a high-risk group for child neglect and maltreatment. Previous findings suggest that early interventions can reduce maltreatment by improving the quality of mother-child interaction, particularly maternal sensitivity. The aim of the current study is to evaluate the effects of a mother-child intervention program using home visits and video-feedback regarding mother-child interaction (STEEP-b) plus psychiatric treatment of the mother in cases where mental illness is present compared with TAU (treatment as usual, that is, standardized support by the child welfare system) on enhancing maternal sensitivity and child responsiveness in adolescent, high-risk mothers. The second aim of the current project is to investigate behavioral and neural differences between adolescent and adult mothers at baseline and postintervention.

**Methods/Design:**

This is a randomized controlled trial (RCT) with 120 high-risk adolescent mothers (<21 years old) and their 3- to 6-month-old children. Half of the participants will be randomized to receive STEEP-b in addition to their standard treatment for up to 12 to 18 sessions over 9 months. The other half will continue with treatment as usual. For further comparisons, 40 adult mothers with positive and negative rearing experiences (>25 years) will additionally be recruited to investigate behavioral and neural differences between the adolescent and adult group. Blind assessments will take place at T1 (pre-intervention), at the end of the 9-month intervention (T2, postintervention) and 6 months postintervention (T3, follow-up). Moderators of treatment outcomes and sociodemographic data will be assessed at T1. The primary outcome hypothesis is that STEEP-b added to treatment as usual will improve maternal sensitivity and child responsiveness compared with treatment as usual alone in high-risk adolescent mothers. The primary hypothesis will be evaluated at the end of the 9-month follow-up assessment based on the intention-to-treat principle. The trial is funded by the German Ministry for Research and Education (BMBF). Data collection started in October 2012.

**Discussion:**

This is a randomized controlled trial that evaluates the effects of an early intervention program (STEEP-b) on the quality of mother-child interaction and child development in adolescent, high-risk mothers.

**Trial registration:**

DRKS00004409 (27 September 2012)

## Background

Child maltreatment is a pervasive social problem with devastating long-term effects for the child’s later development [1]. Maltreated children show more externalizing behaviors and internalizing problems [2, 3]) and poorer cognitive and school functioning [4, 5]. Attachment insecurity, particularly disorganization, is also highly prevalent among maltreated children [6], which may explain the increased risk for psychopathology later in life [7]. The prevalence of childhood maltreatment, including physical abuse, neglect, sexual abuse, or psychological abuse of children, is estimated to range between 10 and 15 % in the overall German population [8, 9]. An estimated 20 to 30 % of maltreated children are likely to become abusive parents themselves [10]. Given the high burden of child maltreatment, the transmission of maltreatment across generations is a major challenge for our society. Hence, to stop this cycle of violence, a better understanding of this intergenerational transmission along with effective interventions are urgently needed.

Previous studies indicate that mothers with a history of child maltreatment show a less sensitive and empathic parenting style and increased hostility towards their child [2–4], which is supported by several empirical studies showing that low maternal sensitivity correlates with child abuse and neglect [11–13]. Attachment theory [14] proposes that based on the emotional quality of the parent-child relationship, particularly parental sensitivity during infancy and early childhood, individuals develop internal working models of their relationship experiences that influence their parenting behavior in later life. Therefore, low maternal sensitivity, a key factor for the development of attachment security, may contribute to the transmission of maltreatment across generations.

In addition to social learning and attachment theories, new insights into the mechanisms underlying the intergenerational cycle of abuse come from recent neurobiological investigations. For example, childhood maltreatment might involve epigenetic changes that are, for example, related to stress hormone receptors in the brain [15], thereby leading to long-term alterations in neuroendocrine and brain development [16], in particular in prefrontal-limbic and reward pathways that are associated with affect regulation and are critical for parenting.

Adolescent mothers present a high-risk group of mothers for child maltreatment and neglect [17]. They have more frequently experienced a history of childhood neglect, physical and sexual abuse and inconsistent parenting than have adult mothers. Adolescent mothers also suffer more frequently than adult mothers from psychiatric disorders, such as postpartum depression, posttraumatic stress syndrome, substance abuse and personality disorders [18]. Early pregnancy in Germany, as in other industrialized countries, is more common among individuals with lower socioeconomic status and poor education [19]. All of these factors are also associated with an enhanced risk of child neglect and maltreatment [20]. Thus, previous studies have shown that adolescent mothers show less sensitivity towards the child’s needs, have more instrumental and less vocal exchanges and engage in less affectionate behavior than adult mothers [21]. They seem to engage more often in harsh parenting behavior [22] and less in dyadic reciprocity. As a consequence, children of adolescent mothers have a higher risk of disturbed cognitive and emotional development, and they display more externalizing and aggressive behavior than children of adult mothers [23]. Thus, children of adolescents present a high-risk group for child neglect and maltreatment compared with children of adult mothers [23], especially if other stressors are present.

The prevalence and consequences of child maltreatment, within and across generations, underscore the need for effective treatment approaches, particularly in high-risk families. Previous studies have shown that early intervention programs focusing on mother-child interaction significantly improved maternal sensitivity, which was associated with a decline in abuse potential [24]. This is supported by a meta-analysis showing that the most effective interventions that promote children’s attachment security have a clear behavioral focus on maternal sensitivity, which supports the idea of a causal role of maternal sensitivity in the development of attachment security [25]. As already outlined, adolescent mothers are a high-risk group for neglecting and abusing their children. However, many intervention programs are not appropriate for high-risk adolescent parents, and adolescent mothers frequently do not participate in traditional programs [26]. Therefore, a home-visiting training program for young mothers was developed that includes sensitivity training and psycho-education (STEEP, Steps toward effective and enjoyable parenting) based on the results of a prospective long-term study on parenting and children’s development (Minnesota study [27]). This program has been evaluated in a group of 58 mother-child dyads in Germany using a nonrandomized design showing that mothers’ parenting styles and depression as well as children’s attachment security (albeit to a lesser degree) could be improved; however, no effect was found for attachment disorganization [28]. One explanation is that maternal sensitivity is predictive of attachment security but not strongly associated with disorganized attachment [29], whereas child maltreatment and harsh or frightening parenting behaviors are associated with disorganized attachment [30]. Preventing the development of disorganized attachment is particularly important because attachment disorganization is associated with problematic long-term outcomes [31–33], suggesting that early intervention programs aiming to improve attachment security and organization should also focus on reducing harsh parenting behaviors.

The aim of the Teenage Mothers Study (TeeMo) - in the frame of the *Understanding and Breaking the Intergenerational Cycle of Abuse* (UBICA) consortium - is to evaluate the effects of a parental program based on STEEP that focuses on enhancing maternal behaviors related to maternal sensitivity and reducing harsh and frightening parenting behaviors. In addition, in cases where a mental disorder is present in the mother, individualized psychiatric treatment of the mother is provided because maternal psychiatric disorders may additionally increase the risk for inadequate parenting and childhood maltreatment [34]. The intervention condition is compared with treatment as usual (TAU, that is, standardized support by the child welfare system) in adolescent, high-risk mothers. Extending previous studies with a randomized controlled trial (RCT) design, we would like to test whether maternal sensitivity and child responsiveness, which reflects a child’s eagerness to respond to their mother and her or his emotional connection to the mother during mother-child interaction; attachment security and organization; and social-emotional, cognitive, motor and language development, can be improved. The second aim of the current project is to investigate neurobiological and behavioral differences pre- and postintervention in adolescent mothers, as well as between adolescent and adult mothers and their children at baseline. Here, exploratory within- and between-group comparisons will involve possible neurohormonal alterations related to the hypothalamic-pituitary-adrenal (HPA) stress axis and the oxytocin system as well as changes in the fronto-limbic emotion regulation network and in reward networks that are critical for parenting.

## Methods/Design

### Trial design

The Teemo study is designed as a single-center, randomized controlled, investigator-blinded superiority trial with two parallel groups and a primary endpoint of maternal sensitivity and child responsiveness after 9 months of intervention. Randomization will be performed as block randomization with a 1:1 allocation. The trial is located at the Department of Child and Adolescent Psychiatry, University Hospital RWTH Aachen.

### Participants

Caucasian mothers 21 years old or younger with children between 3 and 6 months old and who receive support from the local youth welfare system and agree to participate will be included in the study. To increase the number of eligible participants, the study is being advertised in a local newspaper, obstetric clinics, midwife practices and pediatrician practices. Considering drop-out rates of 30 % during the entire study, a minimum of 180 mothers will be included. For further comparisons 40 adult mothers with positive and negative rearing experiences who are at least 25 years old and matched for education will additionally be recruited to investigate behavioral and neuronal differences between the adolescent and adult group at baseline, postintervention and follow-up. Inclusion and exclusion criteria are listed in Table 1.

**Table 1:** Inclusion and exclusion criteria

Inclusion criteria		
	Maternal criteria	21 years old or younger at the beginning of pregnancy
		Mother and child live together
		Sufficient verbal and intellectual abilities to participate in a verbal training program
		Caucasian
		Written informed consent of the mother and, if <18 years old, of the caregiver of the mother
	Child criteria	Between 3 and 6 months old
		Written informed consent of the caregiver
Exclusion criteria		
	Maternal criteria	Current substance abuse
		Current suicidal ideation
		Psychotic disorders
		Separation from the child (>3 months)
	Child criteria	Preterm birth (<36 weeks gestation)
		Serious medical problems
		Genetic syndromes

### Intervention: STEEP-b

The intervention (STEEP-b) is an adaptation of the STEEP program [35], which aims to enhance sensitive care using video-feedback to guide parents to recognize their children’s signals, take their children’s perspective and understand their children’s needs. STEEP-b was designed to be relatively brief, completed in 12 to 18 sessions, and started when the children are between 3 and 6 months of age (as a detailed meta-analysis could demonstrate [25], interventions with a small to moderate number of sessions and those with an older infant are more effective than longer interventions and those with very young infants). Adolescent mothers are visited at home every 2 to 3 weeks by the same adviser for 9 months. Furthermore, an optional group meeting is offered every second month that mothers can attend. The exact number of sessions will depend on clinical appropriateness in the 9-month window. The original STEEP program is not manualized; however, as treatment manualization is considered an important factor in treatment integrity and effectiveness [25, 36], STEEP-b has been modularized with every session focusing on one of four obligatory modules. The four modules emphasize the following elements: child development, maternal sensitivity, frightening and intrusive behaviors and sensitive discipline. Every module should be worked on twice, with the order depending on clinical appropriateness. A promising tool to increase sensitive parenting is video-feedback [25]. A study in adolescent mothers demonstrated positive maternal behaviors and improved affective scores after video feedback of mother-infant interaction, but not in a control group [37]. Therefore, video-feedback of free interaction situations and structured interaction situations will be used to work on the intervention topics. In addition, if the baseline assessment reveals any psychiatric diseases in the mother (for example, depression or PTSD), arrangements will be made for individual psychiatric treatment to be offered by a qualified clinical psychologist or psychiatrist within 4 weeks after the diagnostic assessments. The intervention is discontinued at the participant’s request or if interventions are no longer possible due to the separation of the mother and child (>3 months) or any other serious harm (severe illnesses of either mother or child, death of participants) preventing study participation. Whenever possible, study participants are retained in the trial to enable follow-up data collection and to prevent missing data. Participants who are assigned to the TAU arm are not allowed to receive any intervention using video-feedback.

### Measurement time points

**Fig. 1:**
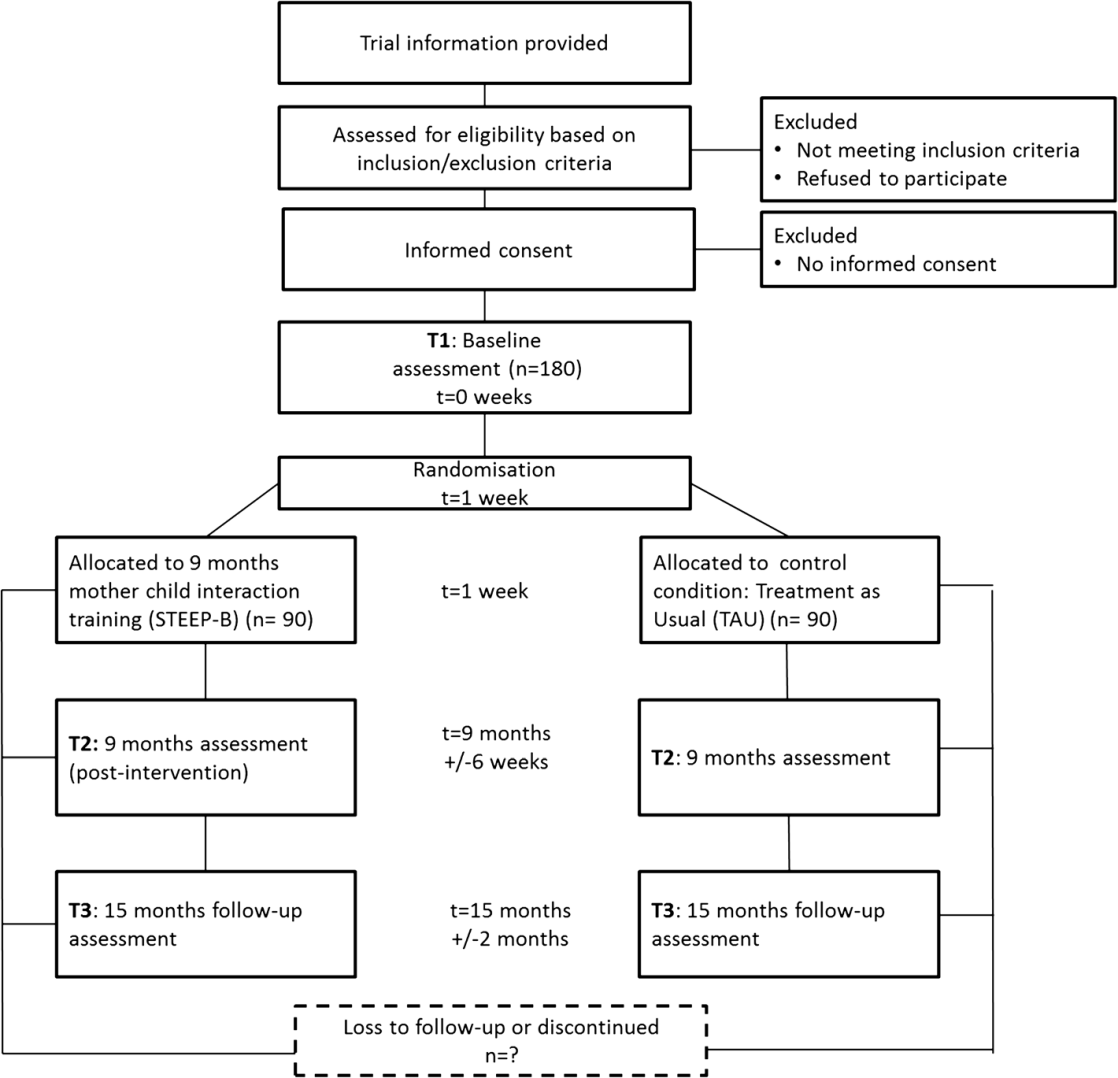
Study flow chart

The flow of participants from recruitment through the end of the study is shown in Fig. 1. Children and mothers will be systematically assessed at T1 (pre-intervention, child age 3 to 6 months), at the end of the 9-month intervention (T2, postintervention) and 6 months postintervention (T3, follow-up). Moderators of treatment outcomes and sociodemographic data will be assessed at T1. Concomitant care is documented at all measurement time points.

Due to the nature of the intervention neither participants nor trainers can be blinded to allocation. Therefore, all follow-up assessments are done by research staff members that are blinded. If unblinding occurs, another assessor will be brought in to re-establish blindness. Participants will be paid 50€ for each assessment session, and travel expenses will be refunded.

### Outcomes

#### Primary outcome measures

The primary outcome variables are maternal sensitivity and child responsiveness as changed from baseline to postintervention. These variables will be assessed by the Emotional Availability (EA) Scales [38–40]. EA is a method to assess the emotional quality of dyadic interactions between an adult and a child. EA has been conceptualized as a research construct derived from attachment theory. It is supported by the empirical literature demonstrating robust links between assessments of attachment and EA in parent- child interactions [41–43]. The EA Scales consist of six dimensions. The adult dimensions are sensitivity, structuring, nonintrusiveness and nonhostility. The child dimensions are responsiveness to the adult and involvement with the adult. All scales range from 1 to 7.

For the assessment of emotional availability, a videotaped free play interaction session as well as a videotaped age-appropriate stress situation session will be evaluated. Maternal sensitivity and child responsiveness are selected as primary outcome variables because of their specific relevance to the development of secure attachment behaviors. Furthermore, they have been shown to be the most reliable EA Scales [44]. Maternal sensitivity refers to the mother’s ability to read and respond to the child’s signals, and child responsiveness reflects a child’s eagerness and willingness to respond to the mother [39].

Videotaped interactions will be rated by psychologists trained for reliability in EA. Raters will be blind with respect to intervention group. Inter-rater reliability of the raters is formally assessed within a subsample of randomly selected videotapes.

#### Secondary outcome measures

Secondary outcome measures for the child are social-emotional, cognitive, motor and language development assessed with the Bayley Scales of Infant and Toddler Development (BSID-III, [45]), social-emotional development assessed with the Brief Infant Toddler Social Emotional Assessment (BITSEA, [46]), child temperament (IBQ-R, [47, 48]; ECBQ, [49]), and child attachment assessed with the Strange Situation Procedure (SSP) [50]. Secondary outcome measures for the mother are overall psychopathological symptoms (BSI-18, [51]), depressive symptomatology (BDI-II, [52]), parental stress (PSI, [53]) and child abuse potential (CAPI, [54]). Furthermore, the emotional quality of the mother-child dyad will be assessed with the following EA dimensions: structuring, nonintrusiveness, non-hostility and child involvement.

At baseline (T1), to examine additional moderators of the outcome measures, we will also ask mothers to complete assessments of intellectual functioning (CFT-20R, [55], an explanation of all abbreviations is given at the end of the text), psychiatric health (MINI, [56]; SKID-II, [57]; STAI, [58]; CAARS, [59]), child abuse history (CECA-Q, [60]), attachment history (VASQ, [61]; ECR-RC, [62]), perception of parental rearing behaviors (EMBU, [63, 64]), impulsivity (BIS, [65]), emotion regulation skills (DERS, [66]) and empathic concern (IRI, [67–69]). Furthermore, sociodemographic data will be collected, including standard questions on age, marital status, household composition, educational level, employment status, characteristics of the child’s father and neonatal data concerning the child and childbirth.

Neurobiological measures will also be assessed to test for moderation effects and for exploratory analyses. To investigate the impact of the hormone oxytocin, plasma oxytocin levels from the mothers will be collected after a standardized period of mother-infant interaction. Furthermore, buccal swabs will be collected from all participants (mothers and children) to investigate the impact of polymorphisms within the glucocorticoid receptor gene (NR3C1), the serotonin transporter gene (SLC6A4), the oxytocin receptor gene (OXTR) and dopamine-system-related genes (DRD4/DRD2/COMT), and epigenetic changes will be assessed by buccal cell samples. To investigate the impact of chronic stress, hair cortisol will be assessed at T1, T2 and T3. For the examination of acute stress, saliva cortisol will be assessed following the SSP in both mother and child. In a subpopulation (*N* = 40 adolescent mothers and *N* = 40 adult mothers), the neural correlates of maternal affect regulation and reward processing will be studied using functional magnetic resonance imaging (fMRI) at T1.

### Sample size calculation and statistical analysis

Based on a large meta-analysis [25], we expect at least moderate effect sizes for maternal sensitivity and child responsiveness (Cohen’s d = 0.5). Assuming a small correlation between baseline and follow-up T2 of 0.25, the standardized effect of d = 0.5 can be detected with a power of 80 % for a two-sided significance level of 5 % with a total sample size of 120 mother-child dyads. Based on Suess *et al*. [28], we expect drop-out rates of approximately 30 % during the longitudinal study; therefore, 180 individuals will be randomized in total to obtain at least 120 evaluable mother-infant dyads. In addition, we expect that approximately 20 % of the screened mothers cannot be included in the study due to violation of the inclusion/exclusion criteria. Hence, 220 to 230 mothers will be assessed for eligibility. If the planned sample size cannot be achieved within the time frame of funding, the Trial Steering Committee and the responsible biometrician of the Data management Team can decide to terminate the trial before the intended sample size is achieved.

All main analyses will be carried out at the end of the last follow up assessment and will be based on the intention to treat principle. Two primary hypotheses, one on the maternal outcome and the other on the child’s outcome, will be tested:

- H_0_: The change from baseline to the 9-month follow-up T2 in maternal sensitivity/child’s responsiveness is equal in the intervention and the control group.
- H_1_: The change from baseline to the 9-month follow-up T2 in maternal sensitivity/child’s responsiveness is different in the intervention and the control group.

An analysis of covariance will be used for primary analyses using the EAS Score at the follow-up T2 as the dependent variable and the baseline value and group allocation as covariates. All other analyses will be performed descriptively. Means and standard deviations or absolute and relative frequencies will be reported according to the underlying scale level. A detailed statistical analytical plan will be written by the trial statistician and signed off by the principle investigator.

### Methods against bias

Eligibility assessment, obtaining informed consent, and enrolling the patient in the trials is performed at the trial site by one of the investigators.

Subjects are randomly assigned to either group using a web-based randomization system (http://www.randomizer.at) in a ratio 1:1. Data entry for randomization is done by the STEEP-b trainer and takes place after all baseline measurements to ensure allocation concealment. The randomization tool is supervised by the Institute of Medical Biometry and Informatics of the University of Heidelberg, Germany. Block randomization using fixed block lengths is used, stratified for mother’s age (<18 years or >18 years).

To ensure that all trainers implement STEEP-b in a very comparable way, all therapists are trained before the beginning of the study, and application of the modularized sessions is strictly monitored. In addition, therapists are supervised by an experienced STEEP therapist (G.J. Suess, STEEP training director in Germany). For the assessments of adherence and competence, sessions will be audiotaped. We will obtain independent judgments on the quality of therapy using a random selection of therapy tapes. Patients will also be asked to assess the intervention and the therapist’s empathy post-intervention.

Data quality is continuously monitored by the Data Management Team (Institute of Medical Biometry and Informatics (IMBI), University of Heidelberg and the Coordination Centre for Clinical Trials Heidelberg (KKS)). Data will be entered electronically at the IMBI. Range checks for data values are done to promote data quality. The original study forms will be kept at the trial site. Participants’ files are stored in locked cabinets and access to the study data will be restricted. Data will be collected, processed and stored according to the data protection laws. The principal investigators will be given access to the cleaned data sets.

### Safety and ethical issues

Research activities within the UBICA-TeeMo project will be carried out in compliance with the fundamental ethical principles as stated by the Declaration of Helsinki. The Coordination Centre for Clinical Trials (KKS) will monitor the study, adapted to ICH-GCP guidelines (E6), and has approved the standard operating procedures. The trial has received approval from the local Research Ethics Committee at the University Hospital RWTH Aachen (reference EK144/12, protocol version 2.0 to 7.0). Any modification to the protocol will require a formal amendment and approval by the local Research Ethics Committee. The protocol must be agreed on by the Trial Steering Committee (principle investigators at the Department of Child and Adolescent Psychiatry, University Hospital RWTH Aachen) and Data Management Team (KKS and IMBI) at the University of Heidelberg.

Participants will be enrolled only after they have received comprehensive information from the responsible investigator. Participants will also receive information sheets and will have the opportunity to discuss the trial with the investigator. Written informed consent for the study will be obtained by the investigator from all participants who are willing to participate in the trial. Additional consent is obtained from participants for collection of genetic and neuroimaging data. Infants will only be included if the adolescent or young adult mother and the guardian have both given informed consent. All participants will be informed that they can stop participating in the study at any time without giving any reasons. Furthermore, additional consent is obtained from all participants who are willing to be contacted by the trial investigator for their participation in ancillary studies.

Study participants are provided with the right to have their information removed from the database at any time. Trial results will be communicated to participants and healthcare professionals who were involved in the study. Results will be published regardless of the magnitude or direction of the effect.

An independent Data and Safety Monitoring Board (DSMB), including two independent clinical experts and one biometrician, is established to monitor the progress of the trial and ensure adherence to protocol. Safety parameters include that all serious adverse events (SAE) or adverse events reported by the subject or detected by the local investigator that occur during the trial and all noticeable problems must be documented in the case report form (CRF). In case of an SAE, the Ethics Committee and DSMB will be informed within 24 h after the SAE becomes known. In case of significant preponderance of SAEs that might causally be associated with the trial, the DSMC will terminate the trial. Patients that are enrolled in the study are covered by indemnity for negligent harm by study insurance policies. Substantive contributions to the design, conduct and interpretation of the trial are recognized through the granting of authorship on the final trial report.

## Discussion

Given the high prevalence of mothers with a history of abuse who maltreat their own children, a better understanding of this intergenerational transmission along with effective interventions to stop this cycle of violence are needed. Thus, there is an urgent need to study the effectiveness of early interventions to improve parental child-rearing attitudes and practices, particularly in at-risk mothers. The main aim of the current study was therefore to investigate within a randomized controlled trial the effects of a home-visiting mother-child intervention program (STEEP-b) plus psychiatric treatment of the mother when mental illness is present compared with TAU (treatment as usual, that is, standardized support from the child welfare system) on maternal sensitivity, child responsiveness, attachment and child development in adolescent, high-risk mothers. Adolescent mothers are a high-risk group for neglecting and abusing their children. Unfortunately, high-risk, adolescent mothers frequently do not participate in traditional intervention programs [26]. Therefore, Erickson and Egeland [35] developed a home-visiting program (STEEP) for young mothers with a focus on maternal sensitivity, a key factor in shaping attachment development and reducing child maltreatment [11–13, 25]. In the current study, this intervention program was adapted (STEEP-b) to improve treatment effectiveness. To this end, the intervention was modularized to enhance treatment integrity [36] with a clear behavioral focus on enhancing maternal sensitivity and reducing harsh parenting behaviors, which may be more effective in shaping attachment security and organization and thereby improving children’s development [25, 29]. Furthermore, the intervention was designed to be relatively brief (12 to 18 sessions) and started when the children are between 3 and 6 months old because a meta-analysis has shown that less, but intensive intervention sessions with an older infant are more effective [25]. In addition, a larger number of adolescent mothers might agree to participate in less frequent, compared with longer, interventions.

Based on the beneficial effects of STEEP on mothers’ parenting styles using a non-randomized design, STEEP is now included in the German “Communities that Care” database as a prevention program with probable empirical evidence. However, to date, no RCT on STEEP has been published, and one is therefore urgently needed.

In the current study, we will be able to investigate behavioral and neurobiological correlates of maternal sensitivity and the impacts of maternal behaviors on a child’s well-being in adolescent and adult mothers. We predict that the intervention will not only improve maternal sensitivity but will also improve a child’s responsiveness to their mother, attachment security, attachment organization and child development. If the current intervention program is successful, the findings will have important implications for community services and clinical work.

## Trial status

Mother-child dyads began to enter the trial in October 2012. Recruitment is still open.

## Abbreviations

BIS-15: Barratt Impulsiveness Scale
BITSEA: Brief Infant-Toddler Social and Emotional Assessment
BDI-II: Becks Depression Inventory, Second Edition
BSI-18: Brief Symptom Inventory
BSID-III: Bayley Scales of Infant and Toddler Development
CAARS: Conners Adult ADHD Rating Scale
CAPI: Child Abuse Potential Inventory
CECA-Q: Childhood Experiences of Care Abuse Questionnaire
CFT 20-R: Culture Fair Intelligence Test - Scale 2, Revision
ECBQ: Early Childhood Behavior Questionnaire
ECR-R: Experiences in Close Relationships Scale-Revised
EMBU: A questionnaire measuring perceptions of parental rearing behaviors
DERS: Difficulties in Emotion Regulation Scale
IBQ-R: Infant Behavior Questionnaire
IRI: Interpersonal Reactivity Index
M.I.N.I: Mini-International Neuropsychiatric Interview
PSI: Parental Stress Index
SCID-II: Structured Clinical Interview for DSM-IV axis II personality disorders
SSP: Strange Situation Procedure
STAI: State-Trait Anxiety Inventory
VASQ: Vulnerable Attachment Style Questionnaire
